# Bis(μ-6-meth­oxy-2-{[(3-oxidoprop­yl)imino]­meth­yl}phenolato)nickel(II) methanol monosolvate

**DOI:** 10.1107/S1600536813017224

**Published:** 2013-06-29

**Authors:** Fan-Kun Meng, Xin Zhang, Hua Yi, De-Hui Zhang, Jun-Ying Jia

**Affiliations:** a Department of Chemistry and Chemical Engineering, Daqing Normal University, Daqing, Heilongjiang 1637121, People’s Republic of China

## Abstract

The mol­ecular structure of the title complex, [Ni_2_(C_11_H_13_NO_3_)_2_]·CH_3_OH, contains two Ni^II^ atoms and two doubly deprotonated 6-meth­oxy-2-{[(3-oxidoprop­yl)imino]­meth­yl}phenolate ligands. The Ni^II^ atoms are each four-coordinated in a distorted square-planar geometry by three O atoms and one N atom derived from the phenolate ligands. The solvent mol­ecule is linked to the complex mol­ecule by two O—H⋯O hydrogen bonds.

## Related literature

For the structures and potential applications in magnetism and catalysis of metal clusters, see: Long *et al.* (2010 ▶); Mondal *et al.* (2011 ▶). Schiff bases have been widely investigated in this regard, see: Sarwar *et al.* (2011 ▶). For cluster complexes based on Schiff bases, see: Costes *et al.* (1998 ▶); Mondal *et al.* (2011 ▶). 
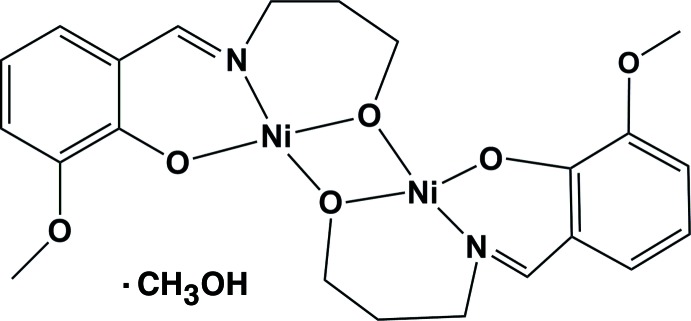


## Experimental

### 

#### Crystal data

[Ni_2_(C_11_H_13_NO_3_)_2_]·CH_4_O
*M* *_r_* = 563.87
Monoclinic, 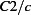

*a* = 23.673 (5) Å
*b* = 8.3124 (17) Å
*c* = 25.546 (5) Å
β = 113.25 (3)°
*V* = 4618.6 (19) Å^3^
*Z* = 8
Mo *K*α radiation
μ = 1.68 mm^−1^
*T* = 150 K
0.26 × 0.24 × 0.22 mm

#### Data collection

Rigaku SCX-mini diffractometer
Absorption correction: multi-scan (*CrystalClear*; Rigaku/MSC, 2002 ▶) *T*_min_ = 0.669, *T*_max_ = 0.709
19153 measured reflections
5257 independent reflections
4285 reflections with *I* > 2σ(*I*)
*R*_int_ = 0.054

#### Refinement

*R*[*F* ^2^ > 2σ(*F* ^2^)] = 0.047
*wR*(*F* ^2^) = 0.080
*S* = 1.01
5257 reflections
312 parameters
1 restraint
H atoms treated by a mixture of independent and constrained refinement
Δρ_max_ = 0.66 e Å^−3^
Δρ_min_ = −0.48 e Å^−3^

### 

Data collection: *CrystalClear* (Rigaku/MSC, 2002 ▶); cell refinement: *CrystalClear*; data reduction: *CrystalClear*; program(s) used to solve structure: *SHELXS97* (Sheldrick, 2008 ▶); program(s) used to refine structure: *SHELXL97* (Sheldrick, 2008 ▶); molecular graphics: *ORTEP-3 for Windows* (Farrugia, 2012 ▶); software used to prepare material for publication: *publCIF* (Westrip, 2010 ▶).

## Supplementary Material

Crystal structure: contains datablock(s) I, global. DOI: 10.1107/S1600536813017224/qm2096sup1.cif

Structure factors: contains datablock(s) I. DOI: 10.1107/S1600536813017224/qm2096Isup2.hkl

Additional supplementary materials:  crystallographic information; 3D view; checkCIF report

## Figures and Tables

**Table 1:** Selected bond lengths (Å)

Ni1—O1	1.9107 (16)
Ni1—O5	1.9208 (16)
Ni1—O2	1.9224 (16)
Ni1—N1	1.9314 (19)
Ni2—O4	1.8936 (16)
Ni2—O2	1.9197 (16)
Ni2—O5	1.9208 (16)
Ni2—N2	1.9366 (19)

**Table 2:** Hydrogen-bond geometry (Å, °)

*D*—H⋯*A*	*D*—H	H⋯*A*	*D*⋯*A*	*D*—H⋯*A*
O7—H7⋯O1	0.86 (1)	2.05 (1)	2.893 (3)	170 (3)
O7—H7⋯O3	0.86 (1)	2.64 (3)	3.178 (3)	122 (3)

**Fig. 1.**
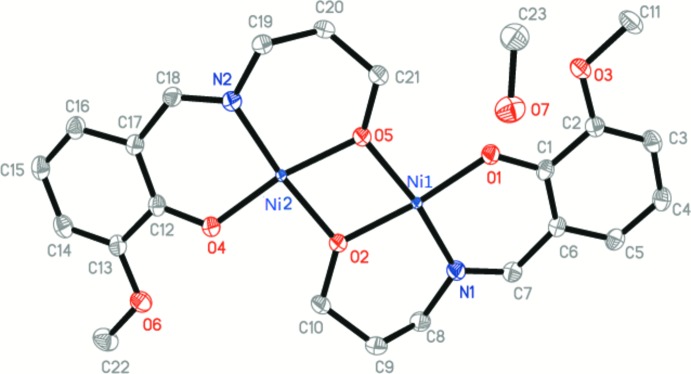
The molecular structure of the title compound. Displacement ellipsoids are drawn at the 30% probability level.

## supplementary crystallographic information

### Comment

Interest in the rational design and synthesis of metal clusters has mushroomed recently, due to their fascinating structures and potential applications in magnetism and catalysis (Mondal *et al.* 2011; Long *et al.* 2010). Schiff bases are widely investigated in this regard (Sarwar *et al.* 2011). Previous reports of a series of 3d cluster complexes based on Schiff bases, have appeared (Mondal *et al.* 2011; Costes *et al.* 1998). Herein, we report a new Ni^II^ complex assembled from the flexible schiff base (2-(((3-hydroxypropyl)imino)methyl)-6-methoxyphenol).

As shown in Fig.1, The molecular structure consists of two Ni^II^ atoms and two doubly deprotonated 2-(((3-hydroxypropyl)imino)methyl)-6-methoxyphenols. The compound crystallized with one molecule of methanol per asymmetric unit. The methanol is hydrogen bonded to O1 and O3. The Ni^II^ atoms are four-coordinated. Four coordination arises from three O and one N atoms derived from two different ligands.The Ni—O distances range from 1.8936 (16)to 1.9208 (16) Å and the Ni—N distances range from 1.9314 (19) to 1.9366 (19) Å, while the O—Ni—O angles range from 76.22 (7) to 168.20 (6)° and the O—Ni—N angles range from 94.61 (8) to 170.39 (8)°.

### Experimental

Treatment of 2-(((3-hydroxypropyl)imino)methyl)-6-methoxyphenol (0.1 mmol, 0.0209 g) with NiCl_2_(0.1 mmol, 0.0238 g) in MeOH (30 ml) gave a green solution. This reaction mixture was stirred for 30 min and then filtered. The solution then stood without perturbation for several days. Green crystals were collected by filtration and air-dried.

### Refinement

H atoms bonded to C were positioned with idealized geometry using a riding model with the aromatic, methylene and methine C—H = 0.948–0.991 Å and the methyl C—H = 0.979–0.981 Å. All H atoms were refined with isotropic displacement parameters set at 1.2Ueq(C-aromatic, methylene and methine) and 1.5Ueq (*C*-methyl).

### Crystal data

**Table:**

[Ni_2_(C_11_H_13_NO_3_)_2_]·CH_4_O	*F*(000) = 2352
*M**_r_* = 563.87	*D*_x_ = 1.622 Mg m^−^^3^
Monoclinic, *C*2/*c*	Mo *K*α radiation, λ = 0.71073 Å
Hall symbol: -C 2yc	Cell parameters from 4060 reflections
*a* = 23.673 (5) Å	θ = 3.0–25.0°
*b* = 8.3124 (17) Å	µ = 1.68 mm^−^^1^
*c* = 25.546 (5) Å	*T* = 150 K
β = 113.25 (3)°	Strip, green
*V* = 4618.6 (19) Å^3^	0.26 × 0.24 × 0.22 mm
*Z* = 8	

### Data collection

**Table:**

Rigaku SCX-mini diffractometer	5257 independent reflections
Radiation source: fine-focus sealed tube	4285 reflections with *I* > 2σ(*I*)
Graphite monochromator	*R*_int_ = 0.054
Detector resolution: 0 pixels mm^-1^	θ_max_ = 27.5°, θ_min_ = 3.0°
ω scan	*h* = −30→30
Absorption correction: multi-scan (*CrystalClear*; Rigaku/MSC, 2002)	*k* = −10→10
*T*_min_ = 0.669, *T*_max_ = 0.709	*l* = −33→29
19153 measured reflections	

### Refinement

**Table:**

Refinement on *F*^2^	Primary atom site location: structure-invariant direct methods
Least-squares matrix: full	Secondary atom site location: difference Fourier map
*R*[*F*^2^ > 2σ(*F*^2^)] = 0.047	Hydrogen site location: inferred from neighbouring sites
*wR*(*F*^2^) = 0.080	H atoms treated by a mixture of independent and constrained refinement
*S* = 1.01	*w* = 1/[σ^2^(*F*_o_^2^) + (0.035*P*)^2^ + 2.8*P*] where *P* = (*F*_o_^2^ + 2*F*_c_^2^)/3
5257 reflections	(Δ/σ)_max_ = 0.008
312 parameters	Δρ_max_ = 0.66 e Å^−^^3^
1 restraint	Δρ_min_ = −0.48 e Å^−^^3^

### Special details

**Table:**

Geometry. All e.s.d.'s (except the e.s.d. in the dihedral angle between two l.s. planes) are estimated using the full covariance matrix. The cell e.s.d.'s are taken into account individually in the estimation of e.s.d.'s in distances, angles and torsion angles; correlations between e.s.d.'s in cell parameters are only used when they are defined by crystal symmetry. An approximate (isotropic) treatment of cell e.s.d.'s is used for estimating e.s.d.'s involving l.s. planes.
Refinement. Refinement of *F*^2^ against ALL reflections. The weighted *R*-factor *wR* and goodness of fit *S* are based on *F*^2^, conventional *R*-factors *R* are based on *F*, with *F* set to zero for negative *F*^2^. The threshold expression of *F*^2^ > σ(*F*^2^) is used only for calculating *R*-factors(gt) *etc*. and is not relevant to the choice of reflections for refinement. *R*-factors based on *F*^2^ are statistically about twice as large as those based on *F*, and *R*- factors based on ALL data will be even larger.

### Fractional atomic coordinates and isotropic or equivalent isotropic displacement parameters (Å^2^)

**Table:**

	*x*	*y*	*z*	*U*_iso_*/*U*_eq_	
Ni1	0.273493 (12)	0.05940 (4)	0.051281 (10)	0.01554 (8)	
Ni2	0.246815 (12)	0.08519 (4)	0.157085 (11)	0.01621 (8)	
O1	0.34489 (7)	0.1069 (2)	0.03665 (6)	0.0228 (4)	
O2	0.21543 (7)	−0.0084 (2)	0.08231 (6)	0.0250 (4)	
O3	0.44965 (7)	0.2040 (2)	0.03304 (7)	0.0304 (4)	
O4	0.17478 (7)	0.0344 (2)	0.16894 (6)	0.0228 (4)	
O5	0.30896 (7)	0.1335 (2)	0.12876 (6)	0.0243 (4)	
O6	0.05845 (7)	0.0169 (2)	0.15204 (7)	0.0312 (4)	
N1	0.22146 (8)	0.0105 (3)	−0.02692 (8)	0.0226 (4)	
N2	0.29151 (8)	0.1639 (2)	0.23379 (7)	0.0216 (4)	
C1	0.34845 (10)	0.1070 (3)	−0.01367 (9)	0.0200 (5)	
C2	0.40454 (11)	0.1544 (3)	−0.01745 (10)	0.0233 (5)	
C3	0.41158 (12)	0.1499 (3)	−0.06854 (10)	0.0274 (5)	
H3A	0.4497	0.1801	−0.0699	0.033*	
C4	0.36276 (12)	0.1009 (3)	−0.11851 (10)	0.0289 (6)	
H4A	0.3680	0.0963	−0.1535	0.035*	
C5	0.30789 (12)	0.0601 (3)	−0.11677 (10)	0.0265 (5)	
H5A	0.2746	0.0300	−0.1509	0.032*	
C6	0.29953 (11)	0.0617 (3)	−0.06476 (9)	0.0216 (5)	
C7	0.23979 (11)	0.0173 (3)	−0.06808 (9)	0.0232 (5)	
H7A	0.2103	−0.0104	−0.1048	0.028*	
C8	0.15658 (10)	−0.0337 (3)	−0.04174 (10)	0.0274 (6)	
H8A	0.1390	−0.0773	−0.0810	0.033*	
H8B	0.1330	0.0638	−0.0408	0.033*	
C9	0.15020 (11)	−0.1576 (3)	−0.00104 (10)	0.0271 (5)	
H9A	0.1817	−0.2423	0.0054	0.033*	
H9B	0.1093	−0.2090	−0.0187	0.033*	
C10	0.15745 (10)	−0.0860 (3)	0.05594 (10)	0.0260 (5)	
H10A	0.1241	−0.0072	0.0501	0.031*	
H10B	0.1539	−0.1723	0.0812	0.031*	
C11	0.50708 (11)	0.2516 (4)	0.03119 (12)	0.0367 (6)	
H11A	0.5356	0.2844	0.0694	0.055*	
H11B	0.5003	0.3420	0.0048	0.055*	
H11C	0.5246	0.1609	0.0182	0.055*	
C12	0.15950 (10)	0.1012 (3)	0.20764 (9)	0.0205 (5)	
C13	0.09631 (11)	0.0956 (3)	0.20039 (10)	0.0249 (5)	
C14	0.07727 (12)	0.1647 (3)	0.23951 (10)	0.0286 (5)	
H14A	0.0349	0.1636	0.2329	0.034*	
C15	0.11962 (12)	0.2371 (3)	0.28913 (10)	0.0299 (6)	
H15A	0.1062	0.2816	0.3165	0.036*	
C16	0.18039 (12)	0.2430 (3)	0.29772 (10)	0.0272 (5)	
H16A	0.2092	0.2905	0.3316	0.033*	
C17	0.20091 (10)	0.1796 (3)	0.25705 (9)	0.0214 (5)	
C18	0.26536 (11)	0.1982 (3)	0.26820 (9)	0.0224 (5)	
H18A	0.2910	0.2397	0.3045	0.027*	
C19	0.35845 (10)	0.1872 (3)	0.25486 (9)	0.0250 (5)	
H19A	0.3790	0.0810	0.2624	0.030*	
H19B	0.3728	0.2472	0.2913	0.030*	
C20	0.37658 (10)	0.2792 (3)	0.21225 (9)	0.0243 (5)	
H20A	0.3505	0.3763	0.1998	0.029*	
H20B	0.4198	0.3154	0.2316	0.029*	
C21	0.37039 (10)	0.1813 (3)	0.16008 (9)	0.0240 (5)	
H21A	0.3849	0.2464	0.1353	0.029*	
H21B	0.3968	0.0846	0.1722	0.029*	
C22	−0.00543 (12)	0.0121 (4)	0.14189 (13)	0.0403 (7)	
H22A	−0.0279	−0.0487	0.1069	0.060*	
H22B	−0.0216	0.1220	0.1377	0.060*	
H22C	−0.0106	−0.0403	0.1741	0.060*	
O7	0.44563 (9)	−0.0862 (3)	0.11244 (8)	0.0405 (5)	
H7	0.4192 (13)	−0.020 (3)	0.0904 (12)	0.061*	
C23	0.49869 (12)	−0.0019 (4)	0.14902 (11)	0.0402 (7)	
H23A	0.5115	0.0747	0.1266	0.060*	
H23B	0.5320	−0.0785	0.1678	0.060*	
H23C	0.4892	0.0564	0.1779	0.060*	

### Atomic displacement parameters (Å^2^)

**Table:**

	*U*^11^	*U*^22^	*U*^33^	*U*^12^	*U*^13^	*U*^23^
Ni1	0.01219 (14)	0.02092 (16)	0.01395 (13)	−0.00189 (11)	0.00562 (10)	−0.00251 (11)
Ni2	0.01350 (14)	0.02149 (17)	0.01487 (13)	−0.00278 (11)	0.00690 (10)	−0.00266 (11)
O1	0.0187 (8)	0.0319 (10)	0.0189 (7)	−0.0028 (7)	0.0087 (6)	−0.0014 (7)
O2	0.0197 (8)	0.0364 (10)	0.0207 (7)	−0.0093 (7)	0.0099 (6)	−0.0073 (7)
O3	0.0208 (8)	0.0414 (12)	0.0306 (9)	−0.0084 (8)	0.0119 (7)	−0.0045 (8)
O4	0.0189 (8)	0.0287 (10)	0.0233 (8)	−0.0023 (7)	0.0111 (6)	−0.0030 (7)
O5	0.0181 (8)	0.0356 (10)	0.0205 (8)	−0.0074 (7)	0.0090 (6)	−0.0064 (7)
O6	0.0176 (8)	0.0428 (12)	0.0338 (9)	−0.0018 (8)	0.0108 (7)	−0.0027 (9)
N1	0.0189 (9)	0.0256 (11)	0.0217 (9)	−0.0010 (8)	0.0065 (7)	−0.0024 (9)
N2	0.0200 (9)	0.0243 (11)	0.0199 (9)	−0.0019 (8)	0.0074 (7)	0.0003 (8)
C1	0.0233 (11)	0.0175 (12)	0.0201 (10)	0.0039 (9)	0.0095 (9)	0.0018 (9)
C2	0.0242 (12)	0.0207 (13)	0.0268 (11)	0.0003 (10)	0.0119 (9)	0.0015 (10)
C3	0.0299 (13)	0.0256 (14)	0.0337 (13)	−0.0013 (11)	0.0201 (10)	0.0042 (11)
C4	0.0387 (14)	0.0304 (15)	0.0239 (11)	0.0058 (11)	0.0192 (10)	0.0051 (11)
C5	0.0312 (13)	0.0290 (14)	0.0196 (11)	0.0044 (11)	0.0104 (9)	0.0011 (10)
C6	0.0232 (11)	0.0219 (13)	0.0214 (11)	0.0033 (9)	0.0108 (9)	0.0014 (9)
C7	0.0234 (11)	0.0237 (13)	0.0203 (10)	0.0024 (10)	0.0064 (9)	−0.0014 (10)
C8	0.0186 (11)	0.0362 (16)	0.0238 (11)	−0.0024 (10)	0.0046 (9)	−0.0028 (11)
C9	0.0217 (11)	0.0296 (14)	0.0283 (12)	−0.0066 (10)	0.0079 (9)	−0.0047 (11)
C10	0.0181 (11)	0.0352 (15)	0.0255 (11)	−0.0078 (10)	0.0096 (9)	−0.0037 (11)
C11	0.0248 (13)	0.0405 (17)	0.0486 (15)	−0.0074 (12)	0.0186 (11)	−0.0023 (13)
C12	0.0243 (11)	0.0182 (12)	0.0221 (11)	0.0034 (9)	0.0126 (9)	0.0049 (9)
C13	0.0243 (12)	0.0268 (14)	0.0258 (11)	0.0012 (10)	0.0122 (9)	0.0047 (10)
C14	0.0276 (12)	0.0270 (14)	0.0379 (13)	0.0050 (11)	0.0202 (10)	0.0066 (12)
C15	0.0408 (15)	0.0255 (14)	0.0339 (13)	0.0048 (12)	0.0258 (11)	0.0008 (11)
C16	0.0393 (14)	0.0220 (13)	0.0263 (12)	0.0005 (11)	0.0193 (10)	−0.0011 (10)
C17	0.0268 (12)	0.0186 (12)	0.0218 (10)	0.0008 (10)	0.0128 (9)	0.0019 (10)
C18	0.0277 (12)	0.0205 (13)	0.0185 (10)	−0.0030 (10)	0.0087 (8)	−0.0003 (9)
C19	0.0198 (11)	0.0324 (14)	0.0201 (10)	−0.0034 (10)	0.0051 (8)	−0.0005 (10)
C20	0.0190 (11)	0.0282 (14)	0.0247 (11)	−0.0065 (10)	0.0076 (9)	−0.0033 (10)
C21	0.0180 (11)	0.0310 (14)	0.0237 (11)	−0.0059 (10)	0.0087 (8)	−0.0043 (11)
C22	0.0203 (13)	0.0457 (18)	0.0537 (17)	−0.0018 (12)	0.0132 (12)	−0.0038 (15)
O7	0.0350 (11)	0.0377 (12)	0.0408 (11)	0.0060 (9)	0.0065 (8)	0.0008 (9)
C23	0.0272 (14)	0.0532 (19)	0.0359 (14)	0.0087 (13)	0.0081 (11)	0.0026 (14)

### Geometric parameters (Å, º)

**Table:**

Ni1—O1	1.9107 (16)	C9—H9A	0.9900
Ni1—O5	1.9208 (16)	C9—H9B	0.9900
Ni1—O2	1.9224 (16)	C10—H10A	0.9900
Ni1—N1	1.9314 (19)	C10—H10B	0.9900
Ni2—O4	1.8936 (16)	C11—H11A	0.9800
Ni2—O2	1.9197 (16)	C11—H11B	0.9800
Ni2—O5	1.9208 (16)	C11—H11C	0.9800
Ni2—N2	1.9366 (19)	C12—C17	1.415 (3)
O1—C1	1.321 (2)	C12—C13	1.434 (3)
O2—C10	1.422 (3)	C13—C14	1.374 (3)
O3—C2	1.373 (3)	C14—C15	1.404 (4)
O3—C11	1.434 (3)	C14—H14A	0.9500
O4—C12	1.304 (3)	C15—C16	1.368 (3)
O5—C21	1.412 (3)	C15—H15A	0.9500
O6—C13	1.372 (3)	C16—C17	1.411 (3)
O6—C22	1.430 (3)	C16—H16A	0.9500
N1—C7	1.288 (3)	C17—C18	1.445 (3)
N1—C8	1.476 (3)	C18—H18A	0.9500
N2—C18	1.291 (3)	C19—C20	1.524 (3)
N2—C19	1.471 (3)	C19—H19A	0.9900
C1—C6	1.412 (3)	C19—H19B	0.9900
C1—C2	1.425 (3)	C20—C21	1.518 (3)
C2—C3	1.380 (3)	C20—H20A	0.9900
C3—C4	1.402 (4)	C20—H20B	0.9900
C3—H3A	0.9500	C21—H21A	0.9900
C4—C5	1.360 (4)	C21—H21B	0.9900
C4—H4A	0.9500	C22—H22A	0.9800
C5—C6	1.419 (3)	C22—H22B	0.9800
C5—H5A	0.9500	C22—H22C	0.9800
C6—C7	1.431 (3)	O7—C23	1.420 (3)
C7—H7A	0.9500	O7—H7	0.855 (10)
C8—C9	1.513 (3)	C23—H23A	0.9800
C8—H8A	0.9900	C23—H23B	0.9800
C8—H8B	0.9900	C23—H23C	0.9800
C9—C10	1.519 (3)		
			
O1—Ni1—O5	94.29 (7)	C9—C10—H10A	109.5
O1—Ni1—O2	166.63 (7)	O2—C10—H10B	109.5
O5—Ni1—O2	76.22 (7)	C9—C10—H10B	109.5
O1—Ni1—N1	95.36 (8)	H10A—C10—H10B	108.1
O5—Ni1—N1	166.94 (8)	O3—C11—H11A	109.5
O2—Ni1—N1	95.57 (8)	O3—C11—H11B	109.5
O4—Ni2—O2	92.84 (7)	H11A—C11—H11B	109.5
O4—Ni2—O5	168.20 (6)	O3—C11—H11C	109.5
O2—Ni2—O5	76.29 (7)	H11A—C11—H11C	109.5
O4—Ni2—N2	94.61 (8)	H11B—C11—H11C	109.5
O2—Ni2—N2	170.39 (8)	O4—C12—C17	124.8 (2)
O5—Ni2—N2	96.71 (7)	O4—C12—C13	118.4 (2)
C1—O1—Ni1	126.27 (14)	C17—C12—C13	116.8 (2)
C10—O2—Ni2	125.24 (14)	O6—C13—C14	124.7 (2)
C10—O2—Ni1	130.61 (13)	O6—C13—C12	114.2 (2)
Ni2—O2—Ni1	103.45 (8)	C14—C13—C12	121.2 (2)
C2—O3—C11	116.59 (19)	C13—C14—C15	120.9 (2)
C12—O4—Ni2	124.27 (15)	C13—C14—H14A	119.5
C21—O5—Ni2	128.02 (13)	C15—C14—H14A	119.5
C21—O5—Ni1	127.41 (13)	C16—C15—C14	119.4 (2)
Ni2—O5—Ni1	103.47 (7)	C16—C15—H15A	120.3
C13—O6—C22	116.4 (2)	C14—C15—H15A	120.3
C7—N1—C8	116.96 (19)	C15—C16—C17	121.0 (2)
C7—N1—Ni1	123.55 (16)	C15—C16—H16A	119.5
C8—N1—Ni1	119.47 (15)	C17—C16—H16A	119.5
C18—N2—C19	117.29 (19)	C16—C17—C12	120.7 (2)
C18—N2—Ni2	123.11 (16)	C16—C17—C18	117.3 (2)
C19—N2—Ni2	119.60 (14)	C12—C17—C18	122.0 (2)
O1—C1—C6	123.7 (2)	N2—C18—C17	126.5 (2)
O1—C1—C2	119.0 (2)	N2—C18—H18A	116.8
C6—C1—C2	117.2 (2)	C17—C18—H18A	116.8
O3—C2—C3	123.8 (2)	N2—C19—C20	111.77 (18)
O3—C2—C1	114.80 (19)	N2—C19—H19A	109.3
C3—C2—C1	121.4 (2)	C20—C19—H19A	109.3
C2—C3—C4	120.3 (2)	N2—C19—H19B	109.3
C2—C3—H3A	119.8	C20—C19—H19B	109.3
C4—C3—H3A	119.8	H19A—C19—H19B	107.9
C5—C4—C3	119.8 (2)	C21—C20—C19	113.7 (2)
C5—C4—H4A	120.1	C21—C20—H20A	108.8
C3—C4—H4A	120.1	C19—C20—H20A	108.8
C4—C5—C6	121.1 (2)	C21—C20—H20B	108.8
C4—C5—H5A	119.5	C19—C20—H20B	108.8
C6—C5—H5A	119.5	H20A—C20—H20B	107.7
C1—C6—C5	120.1 (2)	O5—C21—C20	111.67 (18)
C1—C6—C7	123.7 (2)	O5—C21—H21A	109.3
C5—C6—C7	116.2 (2)	C20—C21—H21A	109.3
N1—C7—C6	127.3 (2)	O5—C21—H21B	109.3
N1—C7—H7A	116.3	C20—C21—H21B	109.3
C6—C7—H7A	116.3	H21A—C21—H21B	107.9
N1—C8—C9	111.67 (19)	O6—C22—H22A	109.5
N1—C8—H8A	109.3	O6—C22—H22B	109.5
C9—C8—H8A	109.3	H22A—C22—H22B	109.5
N1—C8—H8B	109.3	O6—C22—H22C	109.5
C9—C8—H8B	109.3	H22A—C22—H22C	109.5
H8A—C8—H8B	107.9	H22B—C22—H22C	109.5
C8—C9—C10	112.8 (2)	C23—O7—H7	110 (2)
C8—C9—H9A	109.0	O7—C23—H23A	109.5
C10—C9—H9A	109.0	O7—C23—H23B	109.5
C8—C9—H9B	109.0	H23A—C23—H23B	109.5
C10—C9—H9B	109.0	O7—C23—H23C	109.5
H9A—C9—H9B	107.8	H23A—C23—H23C	109.5
O2—C10—C9	110.77 (18)	H23B—C23—H23C	109.5
O2—C10—H10A	109.5		

### Hydrogen-bond geometry (Å, º)

**Table:**

*D*—H···*A*	*D*—H	H···*A*	*D*···*A*	*D*—H···*A*
O7—H7···O1	0.86 (1)	2.05 (1)	2.893 (3)	170 (3)
O7—H7···O3	0.86 (1)	2.64 (3)	3.178 (3)	122 (3)
